# Transcranial alternating current stimulation can modulate the blink reflex excitability. Effects of a 10- and 20-Hz tACS session on the blink reflex recovery cycle in healthy subjects

**DOI:** 10.1007/s10072-024-07719-x

**Published:** 2024-08-03

**Authors:** Simona Maccora, Pierangelo Sardo, Giuseppe Giglia, Angelo Torrente, Vincenzo Di Stefano, Filippo Brighina

**Affiliations:** 1 Department of Biomedicine, Neuroscience and Advanced Diagnostic (BIND), University of Palermo, Palermo, Sicily Italy; 2 ARNAS Civico, Di Cristina, Via del Vespro 143, 90129 Benfratelli, Palermo, Italy

**Keywords:** Blink reflex recovery cycle, Transcranial alternating current stimulation, Beta oscillations, Basal ganglia

## Abstract

**Background:**

The blink reflex excitability, assessed through paired electrical stimuli responses, has been modulated using traditional non-invasive neurostimulation techniques. Recently, transcranial Alternating Current Stimulation (tACS) emerged as a tool to modulate brain oscillations implicated in various motor, perceptual, and cognitive functions. This study aims to investigate the influence of 20-Hz and 10-Hz tACS sessions on the primary motor cortex and their impact on blink reflex excitability.

**Materials and methods:**

Fifteen healthy volunteers underwent 10-min tACS sessions (intensity 1 mA) with active/reference electrodes placed over C4/Pz, delivering 20-Hz, 10-Hz, and sham stimulation. The blink reflex recovery cycle (BRrc) was assessed using the R2 amplitude ratio at various interstimulus intervals (ISIs) before (T0), immediately after (T1), and 30 min post-tACS (T2).

**Results:**

Both 10-Hz and 20-Hz tACS sessions significantly increased R2 ratio at T1 (10-Hz: *p* = 0.02; 20-Hz: *p* < 0.001) and T2 (10-Hz: *p* = 0.01; 20-Hz: *p* < 0.001) compared to baseline (T0). Notably, 20-Hz tACS induced a significantly greater increase in blink reflex excitability compared to sham at both T1 (*p* = 0.04) and T2 (*p* < 0.001).

**Conclusion:**

This study demonstrates the modulatory effect of tACS on trigemino-facial reflex circuits, with a lasting impact on BRrc. Beta-band frequency tACS exhibited a more pronounced effect than alpha-band frequency, highlighting the influential role of beta-band oscillations in the motor cortex on blink reflex excitability modulation.

## Introduction

Brainstem interneuronal excitability can be investigated by recording the recovery cycle of the blink reflex (BRrc). When two electrical stimuli of equal intensity (first stimulus or Conditioning, second stimulus or Test) are delivered to the supraorbital nerve, the second R2 response or R2 test amplitude is influenced by the interstimulus interval (ISI). Particularly, when the ISI is short (shorter than 200 ms), the R2 test is inhibited and gradually recovers with longer ISIs (longer than 500 ms) [1]. Brainstem reflexes can be functionally abnormal in some neurodegenerative diseases, underlying dysfunction of cortico-thalamic, basal ganglia, and brainstem loops [2]. In disorders characterized by dopaminergic lack such as Parkinson's disease (PD) and cranio-cervical dystonias, there is evidence of increased excitability of blink reflex measured by the recovery cycle [1, 3–6]. In blepharospasm where the hyperexcitability of BRrc is considered as one the most consistent finding [7], it has been suggested that an altered influence of the sensorimotor cortices on the basal ganglia and brainstem could play a role in the pathophysiology of dystonia in addition to dopaminergic dysfunction [8]. Taken together, these evidences lead to the hypothesis of a direct influence of basal ganglia on BRrc excitability [3]. Crucially, in line with this data, enhancement of blink reflex excitability has been already demonstrated in patients with juvenile myoclonic epilepsy, prompting disinhibition of cortico-thalamic pathways involved in the excitability of brainstem circuits [9]. Moreover, in animal models, the beta-band (16 Hz) stimulation of the subthalamic nucleus enhanced blink reflex excitability in normal rats as well as in 6-hydroxydopamine induced model of PD and human patients with PD [10].

Several studies have reported a modulating role of different non-invasive brain stimulation (NIBS) techniques on brainstem excitability in healthy subjects and patients. In 2009, De Vito et al. [11] showed that subthreshold low frequency (1-Hz) repetitive transcranial magnetic stimulation (rTMS) reduced blink reflex excitability in a group of 10 healthy volunteers: long-lasting reduction of blink reflex recovery cycle was interpreted as the consequence of reduced cortical excitability and therefore reduced cortico-nuclear facilitation of brainstem interneuronal circuitry. In another study by Cabib et al. (2016), transcranial direct current stimulation (tDCS) was used as NIBS technique able to modify membrane polarization and modulate the probability to generate action potentials [12]. Anodal (excitatory) tDCS over central cortices induced a persistent increase of BR excitability, also evident 10 min after stimulation, with a larger ipsilateral than contralateral effect. Authors hypothesized that these effects could underlie a modulatory effect of tDCS on descending cortico-nuclear pathways, as indirectly suggested by increased facilitation of R1 in case of unilateral hemispheric damage [13]. However, the same authors also reported the ability of constant electrical currents to sensitize trigeminal neurons, as proved by the mild cutaneous sensation induced by the stimulation [12]. Low-frequency rTMS (inhibitory) over the anterior cingulate cortex has been demonstrated to reduce the blink reflex hyperexcitability and was associated with a clinical improvement in patients with blepharospasm [14].

Transcranial alternating current stimulation (tACS) is a relatively new NIBS device, that uses two electrodes placed on the scalp, with the electrodes alternating as the anode and cathode and creating an alternating direction of current flowing through the target region; unlike TMS or tDCS, tACS can modulate brain oscillations. Brain oscillations are the rhythmic patterns of electrophysiological activity in the neural tissue, naturally occurring in the brain, that can be revealed by EEG analysis. Different frequency bands, like theta (4–8 Hz), alpha (8–13 Hz), beta (13–30 Hz) and gamma (≥ 30 Hz) have been related to different functions of the human brain involved in specific tasks [15].

Two main mechanisms have been suggested to explain tACS effects, entrainment and spike-timing dependent plasticity (STDP). Entrainment is the synchronization of two oscillatory systems occurring when a driving external oscillatory force coordinates another oscillating system [16, 17]. The STDP proposed by Zaehle et al. (2010) refers to the ability of different frequencies of tACS to induce long-term potentiation (LTP) or depression (LTD) [18]: in particular, if a neuron is stimulated at the same or lower frequency of its endogenous frequency, the alternating current would lead to potentiation; conversely, if higher stimulation frequencies than the endogenous ones are used, a post-synaptic spike delivered from external stimulation will arrive before the pre-synaptic spike, weakening of the synapse, by means of an LTD-like mechanism.

In this study, we evaluate the effect of alternating currents at different frequency ranges (10-Hz or alfa band and 20-Hz, beta band) on the blink reflex excitability as tested by BRrc in a group of healthy subjects. Given that it is not known whether tACS-induced modulation depends on local activity variations or involves broader networks, the primary aim of this study is to understand if alternating currents can influence subcortical structures. Secondarily, our objective is to verify if a beta-band frequency (20-Hz) stimulation over the sensori-motor cortex can increase blink reflex excitability, similarly to patients with PD or blepharospasm and in animal models undergoing a beta-band deep stimulation of the subthalamic nucleus.

## Material and methods

### Subjects

We initially recruited 17 healthy volunteers; 2 of them were excluded: 1 refused to complete the entire experimental procedure and 1 started a steroid treatment for medical issues some days after the first stimulation session. We finally enrolled a group of 15 healthy subjects (mean age ± SD: 27.4 ± 2.7, 11 females), all right-handed, as assessed by Edinburgh Inventory [19]. None of the participants suffered from any systemic or neurological disorders, as assessed by a clinical neurologist, female subjects were not examined during the menstrual phase (from 5 days before to 5 days after menstruation); none of them was taking any drug known to alter neuromuscular excitability or any medical therapy for three months before the inclusion. All subjects gave written informed consent before enrollment and the study was approved by the Ethical Committee of the University of Palermo and conducted in accordance with the Declaration of Helsinki. All subjects had to fill out a specific form to detect any adverse reaction after the stimulation [20].

### Blink reflex and blink reflex recovery cycle

During the study subjects laid down supine, with their eyes gently closed, on a comfortable examination bed, in a quiet and dimly lit room. Ag–AgCl surface recording electrodes were placed over the orbicularis oculi muscle of both sides (mid-lower eyelid and temple). In all volunteers, the cathode of the stimulating electrode was placed over the right supraorbital notch and the anode 3 cm away, over the skin of the frontal bone. Skin impedance was lower than 5 kΩ. The ground electrode was placed over the nasion. Blink reflex was recorded with a KeyPoint Electromyographic System and was obtained from stimulation of the right supraorbital nerve. The duration of the stimulus was 0.2 ms and the stimulus intensity was set to three times the intensity (mean ± SD: 11 ± 5 mA) needed to obtain a reproducible ipsilateral R2 with an amplitude of at least 50 μV in five consecutive trials. This intensity was maintained constant for all the experimental procedure.

To obtain BR recovery curves, we used the original technique described by Kimura [1]. Briefly filter settings were 20 Hz-10 kHz. We only considered the R2 ipsilateral to the side of stimulation (right supraorbital nerve). We delivered paired stimuli at a constant current at interstimulus intervals (ISI) of 100, 150, 200, 300, 400, 500 e 750 ms. At each ISI, recordings were repeated 4 times at random intervals of at least 20–40 s to avoid habituation. Data were analyzed offline. The amplitude (μV) of R2 responses was measured after the first (conditioning) and the second (test) stimulus. The outcome measure was the R2 amplitude ratio (R2AR), calculated as follows: R2AR = (R2 test amplitude)/(R2 conditioning amplitude) × 100.

### Transcranial alternating current stimulation (tACS)

Participants were seated on a comfortable chair in a dimly lit room. tACS was applied at a fixed intensity of 1 mA delivered by a DC stimulator (Brainstim, EMS, Bologna, Italy). We used saline-soaked sponge electrodes (5 × 7 cm) and flexible elastics to fixate the electrode on the head. The center of the active electrode was placed over C4 (with the long axis anterior–posterior) and the reference electrode over Pz according to the International 10–20 EEG System, this placement was associated with a lower risk of flickering sensations [21, 22]. During real or sham stimulation, impedance was kept below 10 kΩ. Every subject underwent three types of stimulation for 10 min: a. alpha-band stimulation at a fixed frequency of 10 Hz with no direct current offset; b. beta-band stimulation at a fixed frequency of 20 Hz with no direct current offset; c. sham stimulation (20 Hz) with the current turned on for 30 s, with 5 s of fade-in and fade-out, and then turned off. The order of conditions was randomized across participants and all sessions were separated by ≥ 3 days. Participants were blinded to the condition.

### Blinding

We aimed at a double-blind design with respect to the tACS frequency (alpha, beta, sham). To this purpose, a main experimenter who interacted with the participants was unaware of the stimulation frequency and a second operated the tACS device. At the end of all three stimulation blocks (10 Hz, 20 Hz, sham), all volunteers filled out a questionnaire about side sensation felt during tACS sessions. We used the Italian version of the questionnaire by Fertonani et al. (2015), including items about skin sensations (itching, burning, pain, warmth/heat, pinching), metallic/iron taste, fatigue, other (e.g. phosphenes), besides their duration and localization [20]. For every side effect recorded, patients were asked to rate the unpleasant sensation (none = 0, mild = 1, moderate = 2, considerable = 3, strong = 4). According to the questionnaire employed, we also asked the participants to guess whether active or sham stimulation was delivered in each of the three stimulation sessions.

### Protocol setup

**Fig. 1:**
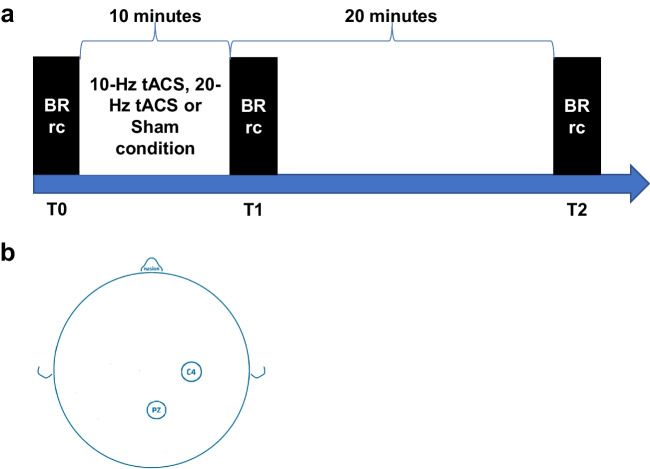
Experimental design. (**a**) Blink reflex recovery cycle (BRrc) was registered before tACS and immediately after 10-min stimulation and 20 min after the end of stimulation (**b**) Stimulating electrodes were positioned over C4 and Pz

Blink reflex recovery cycle was obtained before the stimulation sessions (T0), immediately after 10-min stimulation (T1), and 20 min after the end of each stimulation session (T2) for every stimulation condition (10 Hz, 20 Hz, sham). The timeline of the experimental procedure is represented in Fig. 1.

### Statistical analyses

Onset latencies and amplitudes were pooled to obtain mean values and standard deviations. We built the excitability recovery curves by plotting the mean R2AR against the interstimulus interval for a graphic representation of the effects. To evaluate different stimulation types and times effects, t-tests were performed using the statistical software R. P-values were corrected using Bonferroni post-hoc method, and the significance level was set to 0.05, considering significant tests with p-value < 0.05.

## Results

Latencies of BR components were normal in our sample (R1: 10.1 ± 1.4 ms; ipsilateral R2: 27.2 ± 2.1 ms; contralateral R2: 28 ± 2.1 ms).

**Fig. 2:**
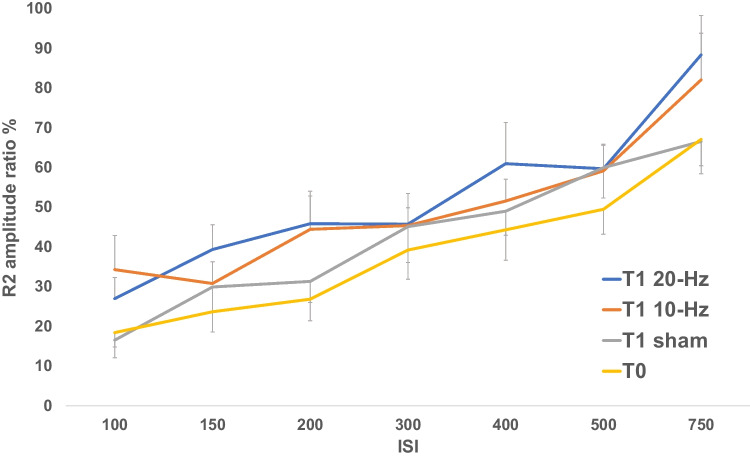
Blink reflex recovery curves at T0 and T1 (after the end of each stimulation session)

**Fig. 3:**
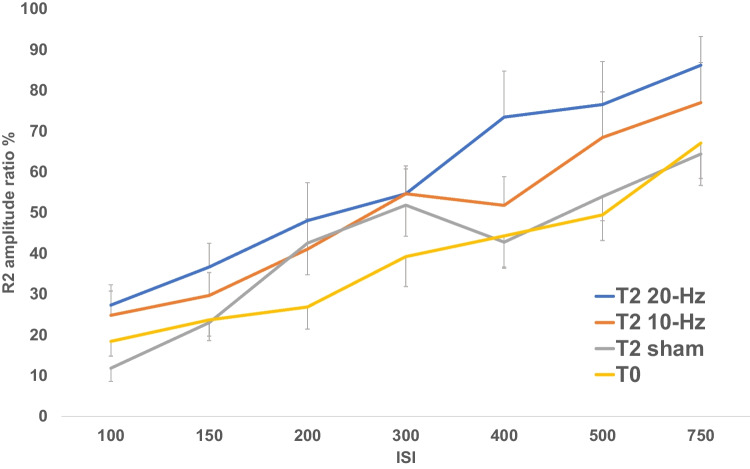
Blink reflex recovery curves at T0 and T2 (20 min after the end of each stimulation session)

Blink reflex recovery curves at T1 are shown in Fig. 2 and at T2 in Fig. 3.

Table 1 refers to 10 Hz, 20 Hz, and sham at T1. It shows the t-test results for the comparisons between T0 and, respectively, 10 Hz, 20 Hz, and sham tACS, between 10 Hz and respectively, 20 Hz and sham, and the comparison between 20 Hz and sham. Specifically, the table shows p-values, the estimated difference between the two comparing groups, and the correspondent confidence interval at 95% level of confidence. Significant tests are shown in bold.

**Table 1:** t-tests results after 10-Hz, 20-Hz, and sham tACS at T1. The table shows *p*-values, the estimated difference between the two comparing groups, and the correspondent confidence interval at 95% level of confidence. Significant tests are shown in bold

	*P*-value	Estimate CI (95%)
T0		
10 − Hz T1	0.02	− 12.29 (− 19.67, − 4.89)
20 − Hz T1	< 0.001	− 14.60 (− 20.12, − 9.08)
Sham T1	1	− 5.34 (− 11.06, 0.39)
10 − Hz T1		
20 − Hz	1	− 2.32 (− 10.38, 5.74)
Sham	0.72	7.30 (0.34, 14.26)
20 − Hz T1		
Sham	0.04	9.98 (3.61, 16.34)

*CI* confidence interval, *tACS* transcranial Alternating Current Stimulation

Results show a significant difference between T0 and both 10-Hz and 20-Hz stimulation in T1, but not sham. At T0, R2 ratio is lower than after alpha and beta-band tACS. A small difference is also detected in T1 between 20-Hz and sham, meaning that 20-Hz tACS significantly increases blink reflex excitability when compared to sham.

Table 2 shows the results at T2. At T2 (20 min after the end of stimulation) 10-Hz and 20-Hz tACS significantly increase the R2 amplitude ratio when compared to T0. Moreover, the mean difference between 20-Hz and sham becomes stronger, with a very low *p*-value (*p* < 0.001).

**Table 2:** t-tests results after 10-Hz, 20-Hz, and sham tACS at T2. The table shows *p*-values, the estimated difference between the two comparing groups, and the correspondent confidence interval at 95% level of confidence. Significant tests are shown in bold

	*P* − value	Estimate (IC 95%)
T0		
10 − Hz T2	0.01	− 13.43 (− 20.56, − 6.31)
20 − Hz T2	< 0.001	− 19.75 (− 26.71, − 12.78)
Sham T2	1	− 3.67 (− 8.63, 1.28)
10 − Hz T2		
20 − Hz	1	− 6.31 (− 15.61, 2.98)
Sham T2	0.08	9.76 (3.14, 16.39)
20 − Hz T2		
Sham T2	< 0.001	16.08 (8.92, 23.23)

*CI* confidence interval, *tACS* transcranial Alternating Current Stimulation

The single three frequencies conditions (10 Hz, 20 Hz, and sham) have been also compared to themselves at different times (T1 vs T2), but none of these comparisons showed any significant difference.

A more generic analysis was also carried out, in which the Baseline R2 ratio was compared to the mean R2 ratio in T1 and T2, respectively at 10 Hz, 20 Hz, and sham. This analysis (Table 3) confirmed what has already been seen before, that is 10-Hz and 20-Hz tACS increase R2 ratio when compared to T0.

**Table 3:** t-tests results for comparisons between Baseline and mean R2 ratio in 10 Hz, 20 Hz, and sham stimulation session

	*P*-value	Estimate (CI 95%)
T0		
Mean 10 − Hz	0.003	− 12.86 (− 19.31, − 6.40)
Mean 20 − Hz	< 0.001	− 17.17 (− 22.62, − 11.73)
Mean Sham	0.81	− 4.76 (-9.42, − 0.10)

*CI* confidence interval

To evaluate successful blinding, we used a specific questionnaire after the end of each stimulation session. The questionnaire revealed that 11 participants (73%) considered 20-Hz tACS as the active, 10 participants (66%) stated that sham tACS was the real stimulation and 9 participants marked as active the 10-Hz tACS (60%): χ^2^(2, *n* = 15) = 4.67, *p* = 0.09.

tACS-induced sensations were also recorded and only skin sensations and phosphenes were reported by participants. Both skin sensations and phosphenes began with the stimulation and stopped quickly. 12 participants perceived skin sensations as itching (80%) during real (*n* = 12, 80%) and sham stimulation (*n* = 11, 73%); intensity was rated as mild for 11 participants and moderate in 1 patient receiving 10-Hz and in another patient receiving 20-Hz tACS. The Friedman test showed no significant differences across the three different stimulation sessions: χ^2^(2) = 0.22, *p* = 0.89. 2 participants complaint of mild phosphenes during the stimulation: 1 subject presented with right flickering sensation during 20 Hz tACS, 1 subject noticed of bilateral phosphenes during both 10 and 20 Hz tACS. Even in this case, the Friedman test showed no difference between the three different conditions: χ^2^(2) = 3.00, *p* = 0.22. These two final analyses confirmed a successful blinding.

## Discussion

Our results suggest that tACS may modulate trigemino-facial reflex excitability, as shown by the increased R2AR after 10-Hz and 20-Hz tACS compared to baseline measurements (T0). This effect seems to be specific as evidenced by the inability of sham stimulation to increase BR excitability. Until now, few studies have evaluated the effect of NIBS on blink reflex excitability and notably, on BRrc. In patients with blepharospasm, low frequency (0.2 Hz) repetitive transcranial magnetic stimulation (rTMS) over anterior cingulate cortex can inhibit blink reflex recovery [14, 23]. Another rTMS study conducted by De Vito et al. (2009) showed that low frequency rTMS over primary motor cortex could reduce the excitability of BR in 10 healthy subjects [11]. No change in BRrc was described after high frequency (20-Hz) rTMS over primary motor cortex in a group of healthy subjects and patients with spinal cord injury [24]. In a tDCS study, increase of BR recovery was reported after a single tDCS session over central areas in case of uni-hemispheric and bi-hemispheric stimulation [12], thus supporting previous neuroimaging studies that suggested that anodal direct currents could modulate nervous structures, distant from stimulation site [25]. Other studies used different BR paradigms to test the potential effect of NIBS on BR as in a study by Suppa et al. (2014) where intermittent theta burst stimulation (iTBS) increased the R2 component whereas continuous TBS decreased it [26].

To our knowledge this is the first study using alternating currents known to modulate cortical oscillations and evaluating their effect on the excitability of brainstem circuits. Both alpha- and beta-band stimulations increased the blink reflex excitability and this seems to suggest the possibility to disrupt brainstem interneuronal excitability, by modifying the cortical oscillations. Indeed, the existence of networks involving the sensorimotor cortices, basal ganglia, thalamus and brainstem has been considered in the pathophysiology of some extrapyramidal disorders. In blepharospasm, for instance, Peterson DA and Sejnowski TJ (2017) hypothesized that apart from an alteration of the nigrostriatal system, blink reflex hyperexcitability depends on motor cortex dysfunction affecting basal ganglia and therefore brainstem but also on altered connections between the trigemino-facial arch, the thalamus and the sensory cortex [8]. This is in line with other studies using voxel-based morphometry (VBM), showing reduction of grey matter volume in sensory-motor and anterior cingulate cortices – areas directly involved in the supranuclear control of blink reflex—in patients with blepharospasm [27].

Our results may reflect two main effects induced by tACS. The first effect could rely on a true entrainment of the sensorimotor cortex induced by tACS, leading to facilitation of cortico-nuclear pathways. Moreover, modulation of ongoing rhythmic activity and entrainment could interfere with the functioning of basal ganglia-thalamus-cortex circuits, also involved in the excitability of BR [28]. As a matter of fact, mu- or rolandic rhythm is an arch-shaped rhythm taking place in the central cortex of normal subjects, comprising two spectral peaks, respectively at 10 and 20 Hz [29, 30]. The levels of these frequencies may vary with voluntary movement: 10-Hz activity is suppressed earlier than 20-Hz activity before movement initiation, whereas 20-Hz increases after the movement is finished [31, 32]. Moreover, the 10-Hz mu rhythm is influenced by tactile stimulation, motor activity and during binocular rivalry [33]. According to this line of evidence, we hypothesize that increased synchronization of both spectral peaks of mu-rhythm induced by alpha- or beta-band tACS can give rise to increased BR excitability in both active tACS conditions. In our experimental paradigm, stimulation site was C4, corresponding to sensorimotor cortex, that has been described as involved in both motor preparation and execution [34], as well as in attentional orienting to a particular time-point [35]. On this basis we can speculate that modulatory effects on BRrc could also be due to an indirect effect on anticipatory attention to sensory stimuli. On this point it is worth to also mention that both alpha and beta bands have been described as involved in different moments of movement selection in sensorimotor cortex, even if at different time points [36]. According to recent findings, we could also hypothesize that alpha spectral frequency of mu could be involved in the processing of somatosensory information whereas the beta frequency directly controls the motor output as testified by a tACS study: Fabbrini et al. (2022) observed an online increased amplitude of N20 only after a session of mu-tACS (frequency 11.7 ± 1.3 Hz) [37].

In our study, only 20-Hz tACS significantly increased the R2 amplitude ratio when compared to sham, suggesting a frequency-dependent potentiation of excitatory cortical drive on interneuronal pool in brainstem. This in agreement with other studies using tACS in which 20-Hz tACS (beta range) increased motor evoked potential (MEP) size [21, 22] and could be explained in our study by an increased activation of corticonuclear fibers. More substained increase of R2 ratio after beta-band tACS could also underlie an increased excitability of hyperdirect pathway, connecting the motor cortex to the subthalamic nucleus [38], suggesting that the modulation of cortical oscillations plays a role in the function of subcortical structures such as those of the long-loop cortex-basal ganglia-thalamus-brainstem circuit. Accordingly, beta-band frequencies are prominent in the motor system and can be recorded not only in somatomotor cortex but also in cerebellum and basal ganglia [39]. In the cortex-basal ganglia circuit, beta activity promotes tonic activity rather than voluntary movement [39, 40]. In support of this hypothesis, beta tACS slowed movement in healthy subjects [41] and it's been already demonstrated that motor impairment in PD could also depend on exaggerated beta-band activity in motor cortex and subthalamic nucleus [42]. In PD, pathological beta hypersyncronization in subthalamic nucleus could partially depend on low dopamine levels as shown by several studies observing a reduction of beta activity after dopaminergic treatment [43]. Increase of BR excitability after 20-Hz tACS is strongly in agreement with a study conducted in animal PD model showing that beta-band (16 Hz) DBS of subthalamic nucleus can lead to blink reflex hyperexcitability [10].

The other modulatory effect by tACS on BRrc could be independent from the frequency of stimulation. As for transcranial direct current stimulation (tDCS), the most frequently reported adverse effect of tACS is itching or burning sensation under the electrodes during the first seconds of stimulation that can be due to trigeminal sensitization [12]. Even in our sample, the majority of subjects (80%) reported slight skin sensations under the electrodes even when sham stimulation was delivered over the scalp. This phenomenon could suggest that also alternating currents could sensitize trigeminal fibers, leading to increased BR excitability as already observed for tDCS [12]. However, in our sample, no significant difference in skin sensation was detected between real and sham sessions, indicating that tACS effect on BRrc chiefly depends on frequency of stimulation and doesn’t rely on effects on trigeminal sensitization. Further studies including larger samples could help replicate these results.

Excitability of BR is persistent even 20 min after the end of stimulation, especially after 20-Hz tACS. This is in line with other studies demonstrating that tACS could increase endogenous EEG power in the range of the stimulation frequency also after 30 min [18] and up to 70 min after the stimulation [44] although following studies failed to demonstrate any after-effects of high-current tACS [45]. This could also reflect the other proposed mechanism of tACS, the so-called spike-timing-dependent-plasticity (STDP) [46–48] because the magnitude of synaptic strength relies on the rhythm of electrical stimulation-derived excitation along with the intrinsic oscillatory pattern.

This study has several limits which worth mention. Future studies including a larger number of participants could detect any clear difference between alpha- and beta-band tACS. Moreover, we didn’t explore corticonuclear and corticospinal excitability by motor evoked potentials. We didn’t evaluate online effects because of current artifacts. Further studies are needed to solve these issues. Moreover, we didn’t compare tACS to other NIBS techniques (e.g. rTMS). Despite all these limitations, this is the first study evaluating the role of cortical oscillations in influencing brainstem circuits and we demonstrated that 20-Hz tACS enhances BR excitability in healthy subjects because of entraining of sensorimotor cortex and activation of cortico-subthalamic loops directly involved in the supranuclear control of blink responses.

## Data Availability

The datasets generated and/or analyzed during the current study are available from the corresponding author on reasonable request.
